# Low preoperative total lymphocyte count as a predictor of poor outcome in adult cardiac surgery

**DOI:** 10.1186/cc9833

**Published:** 2011-03-11

**Authors:** V Lomivorotov, S Efremov, V Boboshko

**Affiliations:** 1 Novosibirsk State Institute of Circulation Pathology, Novosibirsk, Russia

## Introduction

Evaluation of operational risk is an important goal of perioperative management of patients in cardiac surgery. The aim of this study was to investigate the prognostic value of preoperative total lymphocyte count (PTLC) in peripheral blood as a predictor of postoperative complications and mortality in cardiac surgery.

## Methods

A retrospective observational study of 1,380 adults who were operated on the heart using cardiopulmonary bypass (CPB) in 2009. Patient characteristics, hospital mortality, postoperative complications, ventilation time, ICU and hospital stay were analysed. Patients were divided into four groups depending on their PTLC: <1,000 cells/μl, 40 patients; 1,000 to 1,500 cells/μl, 199 patients; 1,501 to 2,000 cells/μl, 414 patients; and >2,000 cells/μl, 715 patients. Analysis was performed using univariate analysis, Kruskal-Wallace test or Fisher-Freeman-Halton exact test (for qualitative characteristics). Univariate and multivariate logistic regression analysis of in-hospital mortality also were performed. *P *< 0.05 was considered statistically significant.

## Results

PTLC <1,500 cells/μl was associated with significantly higher mortality by univariate (OR = 3.53; CI = 1.98 to 6.28; *P *< 0.0001) and multivariate (OR = 2.06; CI = 1.02 to 4.15; *P *< 0.044) analysis. Low preoperative total lymphocyte count was associated with more frequent inotropic support (*P *< 0.001); postoperative heart arrhythmia (*P *< 0.001); dialysis-dependent acute renal failure (*P *< 0.001); and a prolonged ventilation time (*P *= 0.001), ICU stay (*P *< 0.001), and hospital stay (*P *= 0.007). Furthermore, patients with low PTLC were readmitted to the ICU more often (*P *= 0.008). There were no intergroup differences in age and body mass index.

## Conclusions

PTLC is an informative, simple and easily reproducible criterion for evaluating the operational risk in cardiac surgery. However, detailed mechanisms responsible for correlations between preoperative PTLC and cardiovascular morbidity and mortality remain unknown.

